# Resting-state connectivity studies as a marker of the acute and delayed effects of subanaesthetic ketamine administration in healthy and depressed individuals: A systematic review

**DOI:** 10.1177/23982128211055426

**Published:** 2021-11-15

**Authors:** Vasileia Kotoula, Toby Webster, James Stone, Mitul A Mehta

**Affiliations:** 1 Centre for Neuroimaging Sciences, Institute of Psychiatry, Psychology and Neuroscience, King’s College London, London, UK; 2 GKT School of Medical Education, London, UK; 3 University of Sussex, Brighton, UK

**Keywords:** Ketamine, resting state, acute ketamine changes, delayed ketamine effects, major depressive disorder

## Abstract

Acute ketamine administration has been widely used in neuroimaging research to mimic psychosis-like symptoms. Within the last two decades, ketamine has also emerged as a potent, fast-acting antidepressant. The delayed effects of the drug, observed 2–48 h after a single infusion, are associated with marked improvements in depressive symptoms. At the systems’ level, several studies have investigated the acute ketamine effects on brain activity and connectivity; however, several questions remain unanswered around the brain changes that accompany the drug’s antidepressant effects and how these changes relate to the brain areas that appear with altered function and connectivity in depression. This review aims to address some of these questions by focusing on resting-state brain connectivity. We summarise the studies that have examined connectivity changes in treatment-naïve, depressed individuals and those studies that have looked at the acute and delayed effects of ketamine in healthy and depressed volunteers. We conclude that brain areas that are important for emotional regulation and reward processing appear with altered connectivity in depression whereas the default mode network presents with increased connectivity in depressed individuals compared to healthy controls. This finding, however, is not as prominent as the literature often assumes. Acute ketamine administration causes an increase in brain connectivity in healthy volunteers. The delayed effects of ketamine on brain connectivity vary in direction and appear to be consistent with the drug normalising the changes observed in depression. The limited number of studies however, as well as the different approaches for resting-state connectivity analysis make it very difficult to draw firm conclusions and highlight the importance of data sharing and larger future studies.

## Introduction

Ketamine is a *N*-methyl-d-aspartate receptor (NMDA) receptor antagonist which is commonly prescribed as an anaesthetic and is also used recreationally. In research, ketamine has been widely used as a model of psychosis in both animal as well as human studies (Frohlich and Van Horn, 2014). Within minutes of administration, subanaesthetic doses of ketamine produce strong dissociative effects that have been described as psychosis-like symptoms (Krystal et al., 2005). Several studies have investigated these acute effects of ketamine in the brain which include widespread increases in metabolism, blood flow and blood oxygen level–dependent (BOLD) signal (Bryant et al., 2019; Lally et al., 2015; Mueller et al., 2018). During the last two decades, ketamine has emerged as a potent, fast-acting antidepressant, currently licenced for Treatment-Resistant Depression (Schwartz et al., 2016; Zarate et al., 2006). The antidepressant effects of ketamine appear to be different compared to the effects produced by commonly prescribed antidepressant medication (Berman et al., 2000). While current antidepressant medications typically take several weeks to produce a detectable clinical effect, ketamine’s antidepressant action has been detected within hours of drug administration and can last on average, up to 1 week after a single infusion (Zarate et al., 2006).

Several studies have focused on the molecular mechanisms that may underlie the antidepressant effects of ketamine in the brain (for review, see Zanos and Gould, 2018). At the systems’ level, the delayed effects that accompany the antidepressant action (2–24 h after infusion) are only now beginning to be understood. Several key outstanding questions need to be addressed in order to comprehend ketamine’s antidepressant effects in the brain. Are the brain areas sensitive to acute drug administration different from those areas that are sensitive to the delayed effects? Are the brain changes produced by ketamine directly targeting areas affected in depression or is ketamine’s antidepressant action demonstrated in a more indirect manner? At the systems’ level, neuroimaging studies are beginning to chart these effects. While important insights may be gained from other modalities, such as positron emission tomography (PET) or task-based magnetic resonance imaging (MRI), this review will focus on the resting-state studies since this methodology has been more widely employed to examine not only the connectivity changes observed in major depressive disorder (MDD) but also the acute and delayed effects of ketamine’s administration in healthy and depressed volunteers.

In this systematic literature review, we aim to (1) summarise reported changes in connectivity in unmedicated patients with depression, (2) present reported effects of ketamine on brain connectivity in healthy participants and (3) report the effects of ketamine on brain activity in patients with depression. For the purpose of this review, we use the term ‘delayed effects’ to describe any post-acute changes in brain connectivity that occur 2 h to 2 weeks after the drug’s administration. When those ‘delayed effects’ concern MDD patients, they also reflect the antidepressant actions of the drug. By describing the patterns of brain connectivity in depression and ketamine’s effects on the healthy and depressed brain, we hope to identify potential systems-level mechanisms for ketamine’s delayed effects which can serve as candidates for understanding rapid-acting antidepressant mechanisms.

## Methods

**Figure 1.**
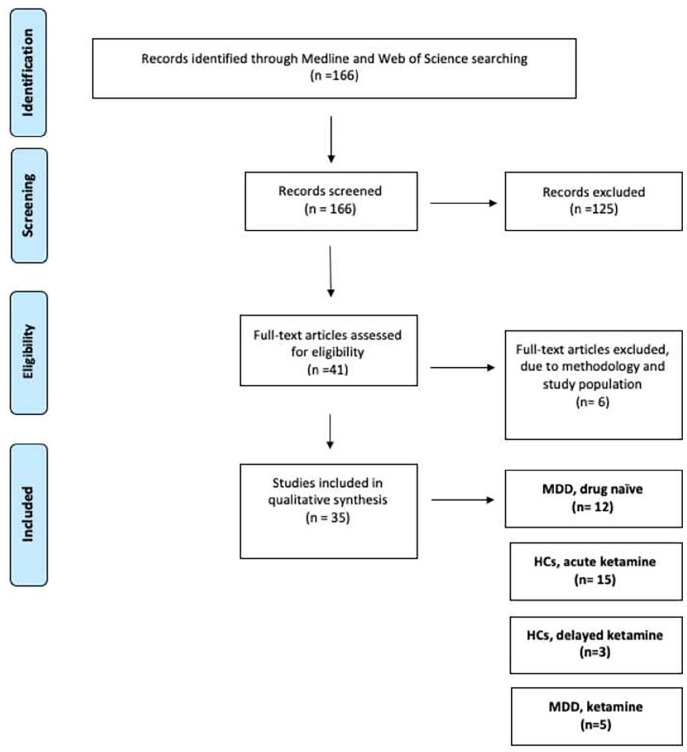
The PRISMA flow diagram shows the process that was followed in order to identify relevant publications for our literature review. From the 166 studies initially identified, 35 fulfil our inclusion criteria and are described in our review.

We followed the PRISMA guidelines for a systematic review and performed a Medline and Web of Science search for articles up to 1 September 2020. Overall, we conducted three main searches. Our first search focused on the identification of studies that have used resting-state MRI to investigate connectivity in treatment-naïve depressed patients. For that purpose, we have used the search terms ‘depression’, ‘drug-naïve’, ‘treatment-free’ ‘resting state’ and ‘imaging’. Studies with participants who were in their in the first-episode depressed and drug-naïve or chronically depressed but with no history of antidepressant treatment were included in our review. A total of 12 studies that fulfilled our research criteria were identified and included in this review. In order to identify studies that examined the acute and delayed effects of ketamine with the use of resting-state MRI, we entered the search terms ‘ketamine’, ‘healthy’, ‘resting state’ and ‘imaging’. We then separated those studies into those that have scanned participants right after the administration of the drug (acute effects) and those that have studied the effects of the drug at least 2 h post its administration (delayed effects). A total of 15 studies looking at the acute effects of ketamine in healthy participants were identified whereas only three studies that reported the delayed effects of ketamine in healthy participants were found. Finally, we searched for studies that have administered ketamine as an antidepressant treatment and have used resting-state MRI to examine the brain changes during the antidepressant effects time window. The terms we have used for this search were ‘antidepressant’ and ‘ketamine’, ‘depression’ and ‘resting state’. A total of five studies fulfilled our research criteria and are included in this review. In addition to our online search, we also used reference lists from the identified articles, reviews and meta-analyses to find additional articles not indexed by PubMed. The number of studies which fulfilled the inclusion criteria of this review was relatively low, and therefore, we decided not to use a sample size criterion in decisions to exclude studies. This allowed a more complete overview of the published literature, but this limitation should be taken into account when the results of those studies are presented, and conclusions are drawn. However, this mainly affected the healthy volunteer studies. We also did not exclude any studies based on the connectivity methodology that they have followed as long as sufficient details about the data processing and analysis were provided in the appropriate section. All the articles in this article were judged by all the authors to meet rigorous scientific standards. Figure 1 provides a summary of all the studies that were identified, screened and selected to be part of this review.

## Results

### Connectivity changes in treatment-naïve MDD patients

#### Subcortical regions of interests

Subcortical brain areas – namely, the ventral caudate (Yang and Wang, 2017), the nucleus accumbens (ΝA) (Gong et al., 2017) and the amygdala (Wei et al., 2016) – were used as seed regions to investigate connectivity changes in depressed patients compared to healthy controls (HCs).

The ventral caudate, bilaterally, showed increased Functional Connectivity (FC) with the right cuneus in the MDD group (n = 40) compared to healthy participants (n = 36). However, the FC of the right ventral caudate with the right middle temporal gyrus and the left ventral caudate with the right superior parietal lobule and right superior frontal gyrus was significantly reduced in MDD patients (Yang and Wang, 2017). When the functional coupling of the reward circuits in patients with MDD (80 patients and 43 HCs) was examined, the right NA presented with decreased positive FC with the left superior temporal gyrus, insular lobe, bilateral middle orbital frontal cortex (OFC), left medial OFC and rostral anterior cingulate cortex (ACC) (Gong et al., 2017). Negative FC between the NA, the bilateral dorsal medial prefrontal cortex (DMPFC) and the left dorsal ACC was also identified in the HC group and was significantly reduced in depression (Gong et al., 2017). The connectivity of the bilateral amygdala with the ventral prefrontal cortex (VPFC) as well as the dorsal lateral prefrontal cortex (DLPFC) was also reduced in the MDD group (49 patients and 50 HCs) (Wei et al., 2016).

#### Cortical region of interests

When the between connectivity of 90 region of interests (ROIs) was examined and compared between depressed and healthy volunteers, several cortical regions presented with increased connectivity in MDD (n = 15) compared to HCs (n = 37). These areas included, the left hippocampus which presented with increased connectivity with the right parahippocampal gyrus, the right inferior frontal gyrus which presented with increased connectivity with the right inferior OFC, as well as the right cuneus that was hyperconnected to the left superior occipital gyrus (Tao et al., 2013). Brain areas that presented with decreased connectivity between the MDD and HC group included the bilateral insula which presented with decreased connectivity with the bilateral putamen. Several brain areas including the superior and inferior frontal gyrus, the precentral gyrus and OFC presented with decreased connectivity with frontal and parietal cortical areas as well as the fusiform gyrus and the precuneus (for more details see Table 1).

**Table 1.** Connectivity studies with MDD, drug-naïve volunteers are summarised in this table based on the connectivity methodology that each study has used. The methodology, aims and hypotheses as well as a brief description of the main findings are included for each study.

Connectivity analysis in treatment-naïve, MDD patients				
Seed-based functional connectivity				
Study	Subjects	Methodology		Summary of results
		Connectivity analysis	Aims/hypothesis	
Yan et al. (2019)	First-episode, drug-naïve MDD patients, n = 318. HC, n = 394. These patients form part of a larger cohort of 1300 MDD patients (826 female).	33 ROIs were selected to examine DMN within-network FC using LMM (Linear Mixed Models) Reproducibility of the analysis assessed by: Using different atlases Finer parcellations Performing meta-analysis instead of LMM Use of global signal regression Use of scrubbing in addition to 24 motion parameters Exploratory network analysis of within and between connectivity included: Visual Network (VN) Sensory Motor Network (SMN) Dorsal Attention Network (DAN) Ventral Attention Network (VAN) Subcortical Network (SN) Frontoparietal Network (FN)	To identify abnormal FC patterns associated with the DMN across cohorts and investigate whether episode type, medication status, illness severity and illness duration contributed to abnormalities.	DMN connectivity First-episode, drug-naïve patients > HC – no significant difference. First-episode, drug-naïve patients > first-episode, on-meds patients – connectivity within the DMN. First-episode, drug-naïve patients > recurrent, on-meds patients – connectivity within the DMN. Exploratory analysis First-episode, drug-naïve patients < HC – connectivity within the VN. First-episode, drug-naïve patients < recurrent, on-meds patients – connectivity between the VN and the SMN and between the SMN and DAN.
Yang and Wang (2017)	First-episode MDD patients, n = 40 (21 males). Three participants were excluded. HC, n = 36 (17 males). Four participants were excluded.	The seeds selected for the FC analysis included: Bilateral Ventral Caudate (VC) Superior Temporal Gyrus (STG) The time course of these seed regions was correlated with the entire brain.	MDD patients would express greater abnormalities in the VC and increased connectivity between the STG and the cuneus and precuneus.	MDD > HC Right VC with the right Occipital Lobe, Cuneus Left VC with the right Occipital Lobe, Cuneus Left STG with the left Parietal Lobe, the Precuneus, the right Angular Gyrus, the left Occipital Lobe and the Cuneus MDD < HC Right VC with the right Middle Temporal Gyrus Left VC with the right Superior Parietal Lobule and the right Superior Frontal Gyrus
Gong et al. (2017)	First-episode MDD patients, n = 80 (33 males). Five participants were excluded. HC, n = 43 (23 males). One participant was excluded.	The left and right Nucleus Accumbens (NA) were used as seeds and examine separately. The time course of the seed regions was correlated with the rest of the brain.	Dysfunctional reward circuits in the NA would contribute to cognitive deficits in depression.	MDD < HC Left NA with the left Bilateral Caudate, the left Medial and Middle Orbital Frontal Cortex, the left Rostral ACC, the left Superior Temporal Gyrus and the Insular Lobe. Right NA with the left Superior Temporal Gyrus, the Insular Lobe, the Bilateral Middle Orbital Frontal Cortex, the right ACC and the left Medial Orbital Cortex. MDD > HC Right NA with the right Bilateral Dorsal Medial Prefrontal Cortex and the left Dorsal ACC.
Guo et al. (2015)	MDD patients, n = 44 (22 males). HC, n = 44 (20 males).	The time course of the bilateral insular cortex seeds was correlated with the rest of the brain.	Altered rsFC of the insula with the rest of the brain between MDD patients and controls.	MDD < HC Right Insula with the left Middle Frontal Gyrus, the left Superior Temporal Gyrus and the right Putamen. Left Insula with the right Middle Occipital Gyrus, the left Superior Temporal Pole and the right Middle Occipital Gyrus.
Guo et al. (2015)	MDD patients, n = 44 (22 males). HC, n = 44 (20 males).	The time course of the Bilateral Crus I was correlated with the rest of the brain.	Increased Crus I-DMN connectivity.	MDD > HC Right Crus I with the right Inferior Frontal Cortex, the right Superior Temporal Pole, the bilateral MPFC and the left Middle Temporal Gyrus.
Cao et al. (2012)	MDD patients, n = 42 (18 males). HC, n = 32 (17 males).	The time course of the Bilateral hippocampal seeds was corrected with the rest of the brain.	Abnormal FC pattern of the hippocampus with the cortical-limbic circuits in MDD.	MDD > HC Left Hippocampus with the Bilateral Middle Frontal Gyrus. Right Hippocampus with the right Inferior Parietal Lobule and the right Cerebellar Tonsil.
ICA (independent component analysis)				
Study	Subjects	Methodology		Summary of results
		Connectivity analysis	Aims/hypothesis	
Zhu et al. (2012)	MDD patients, n = 37 (17 males). Five participants were excluded. HC, n = 37. Four participants were excluded.	ICA was conducted and a template of the DMN was used to examine connectivity changes between the two groups.	Dissociated connectivity pattern between anterior and posterior parts of the DMN.	Within the DMN MDD > HC Dorsal MPFC/Ventral ACC, Ventral MPFC and the Medial Orbital PFC. MDD < HC PCC/Precuneus, Right AG and the left AG/Precuneus.
Veer et al. (2010)	MDD patients, n = 23 (8 males). Four participants were excluded. HC, n = 19 (8 males).	The following ICA networks were identified and compared between the two groups: Primary Visual Network. Lateral Visual Network. Medial Visual Network. Sensory–Motor Network. Right Lateral Network. Left Lateral Network. Precuneus. Ventral Stream Network. Medial Temporal Network. Salience Network. Task Positive Network. Auditory Network. Default Mode Network.	Altered connectivity in the resting-state network (RSN) that includes brain areas associated with affective (ventral PFC, limbic areas) and cognitive (lateral PFC, parietal areas) as well as networks that show corticostriatal connectivity.	Within the Medial Visual Network* (RSN3) MDD < HC Lingual Gyrus with the rest of the network. Within the Auditory Network** (RSN12) MDD < HC Amygdala and Left Insula with the rest of the network and the right Superior Temporal Gyrus. MDD > HC (within the network) Right Inferior Frontal Gyrus. Between the Task Positive (attention and working memory) Network***(RSN11) and the rest of the brain MDD < HC The left Frontal Pole with the rest of the network.
ROI-to-ROI				
Study	Subjects	Methodology		Summary of results
		Connectivity analysis	Aims/hypothesis	
Zhu et al. (2017)	First-episode MDD patients, n = 33 (14 males). Two participants excluded due to head motion. HC, n = 33 (15 males). One participant excluded due to head motion.	The following ROIs of the DMN were defined and included: Anterior medial Prefrontal Cortex (MPFC). Posterior Cingulate Cortex (PCC). Dorsal Medial Prefrontal Cortex (DMPFC) Temporo-parietal junction (TPJ). Lateral Temporal Cortex (LTC). Temporal Pole (TempP). Ventral MPFC. Retrosplenial cortex (Rsp). posterior Inferior Parietal Lobule (pIPL). Parahippocampal Cortex (PHC). Hippocampal Formation (HF). The three DMN subsystems were defined as Midline core: anterior MPFC and PCC. DMPFC subsystem: DMPFC, TPJ, LTC and TempP. MTL subsystem: vMPFC, pIPL, Rsp, PHC, HF.	MDD patients would exhibit altered connectivity in the DMN subsystems.	Between the DMN ROIs Within system connectivity in the DMPFC subsystem MDD > HC – DMPFC-Temp and TPJ-LTC. Inter system connectivity between the DMPFC and Medial Temporal Lobe (MTL) subsystems MDD > HC – TPJ-PHC (inter-system connectivity between the DMPFC and MTL subsystems), LTC-PHC (inter-system connectivity between the DMPFC and MTL subsystems), TempP-vMPFC (inter-system connectivity between the DMPFC and MTL subsystems), TempP-pIPL (inter-system connectivity between the dMPFC and MTL subsystems), TempP-Rsp (inter-system connectivity between the DMPFC and MTL subsystems), TempP-PHC (inter-system connectivity between the DMPFC and MTL subsystems). Within the DMN subsystems MDD > HC – DMPFC subsystem. Between the DMN subsystems MDD > HC – DMPFC subsystem – MTL subsystem.
Wei et al. (2016)	First-episode MDD patients, n = 49 (17 males). HC, n = 50 (17 males).	Correlation analysis between the bilateral amygdala ROI and bilateral PFC mask including Brodmann area 9–12, 24, 25, 32 and 44–47.	Altered connectivity between the amygdala – PFC and amygdala – DLPFC in MDD.	MDD < HC Amygdala – VPFC and DLPFC.
Tao et al. (2013)	First-episode MDD patients, n = 15 (8 males). HC, n = 37 (8 males).	Connectivity analysis The whole brain was parcellated in 90 ROIs based on the anatomical labelling atlas and the time series of these ROIs were compared between the two groups.	To unambiguously identify key connections which are modified in depressed patients.	MDD > HC Left Hippocampus – Right Parahippocampal Gyrus, Right Inferior Frontal Gyrus – Right Inferior Orbitofrontal Cortex, Right Medial Frontal Gyrus – Right Inferior Frontal Gyrus, Right Cuneus – Left Superior Occipital Gyrus and the Right Superior Orbitofrontal Cortex – Right Inferior Orbitofrontal Cortex. MDD < HC Bilateral Insula – Bilateral Putamen, Left Superior Frontal Gyrus – Right Insula, Left Precentral Gyrus – Left Inferior Frontal Gyrus, Right Inferior Frontal Gyrus – Right Supramarginal Gyrus, Left Precentral Gyrus – Left Inferior Parietal Lobule. Right Lingual Gyrus – Right Fusiform Gyrus, Right Angular Gyrus – Right Precuneus and the Left Superior Orbitofrontal Cortex – Left Inferior Orbitofrontal Cortex.
Other connectivity techniques				
Study	Subjects	Methodology		Summary of results
		Connectivity analysis	Aims/hypothesis	
Wang et al. (2012)	First-episode MDD patients, n = 18 (9 males). HC, n = 18 (9 males).	ALFF (Amplitude of Low-Frequency Fluctuations) and fALFF (fractional ALFF) analysis was performed and differences between the two groups were explored.	Altered low frequency amplitude found widely across brain regions linked with PFC, temporal, parietal, occipital and limbic regions.	ALFF results MDD > HC Right Fusiform Gyrus, Right Anterior Lobe of the Cerebellum and the Right Posterior Lobe of the Cerebellum. MDD < HC Left Inferior Temporal Gyrus, Bilateral Inferior Parietal Lobule and the Right Lingual Gyrus. fALFF results MDD > HC Right Precentral Gyrus, Bilateral Fusiform Gyrus, Bilateral Anterior Lobes of the Cerebellum and the Bilateral Posterior Lobes of the Cerebellum. MDD < HC Left Dorsolateral Prefrontal Cortex, Bilateral Medial Orbitofrontal Cortex, Bilateral Middle Temporal Gyrus, Left Inferior Temporal Gyrus and the Right Inferior Parietal Lobule.
Zhang et al. (2011)	First-episode MDD patients, n = 31 (8 males). One participant was excluded. HC, n = 64(30 males). One participant was excluded.	Graph connectivity theory trying to examine the topological organisation of functional brain networks in MDD and compare them to HC.	Disrupted topological organisation of intrinsic functional brain networks.	MDD > HC Decreased path length and increased global efficiency imply a disturbance of the normal global integration of whole-brain networks. Increased nodal centralities were observed in the Caudate Nucleus and the DMN. Decreased nodal centralities were observed in the Temporal Lobes and the Occipital Lobes.

* The Medial Visual Network comprised of areas of the medial occipital cortex.

** The Auditory Network comprised of a functional assembly of regions of the auditory cortex extending into pre- and post-central gyri and more ventral areas such as the insula and temporal poles bilaterally, the media PFC as well as the amygdala.

*** The Task Positive network included the lateral parietal cortex, temporal-occipital junction and the precentral gyrus.

DMN: default mode network; DLPFC: dorsal lateral prefrontal cortex; ACC: anterior cingulate cortex.

The insula bilaterally presented with decreased connectivity in the depressed (n = 44) compared to healthy individuals (n = 44) with the left middle frontal gyrus, left superior temporal gyrus and left superior temporal pole as well as the middle occipital gyrus (Guo et al., 2015). Negative FC was found between the left hippocampus and the bilateral middle frontal gyrus, the right hippocampus and the right inferior parietal lobule as well as the right cerebellum. The magnitude of negative FC was smaller in MDD (n = 42) compared to the control subjects (n = 32) (Cao et al., 2012).

#### Brain networks

Using Independent Component Analysis (ICA), Veer et al. (2010) identified three networks with whose significantly altered connectivity between treatment-naïve depressed individuals (n = 23) and HCs (n = 19). Specifically, within the auditory network, the functional connectivity of the amygdala with the left insula was significantly decreased in the depressed group. For the attention/working memory network, referred to as the task positive network, the connectivity of the frontal poles with this network was also reduced in the MDD group compared to healthy volunteers. Finally, within the visual network, the lingual gyrus was less strongly connected with the rest of the network, in the MDD patients compared to the HCs. The changes in connectivity between and within these networks in depression could relate to some of the emotional as well as cognitive deficits often observed in depressed patients (Veer et al., 2010).

The default mode network (DMN) was the main focus of three resting-state studies since increased connectivity within that network has been linked to depression. A study looking at the overall connectivity within the DMN revealed that compared to HC subjects (n = 37), depressed patients (n = 32) showed increased resting-state functional connectivity within this network. Some decreases, however, were also identified within this network between the posterior cingulate cortex (PCC) and the precuneus/right Angular Gyrus(AG) as well as the left AG and the precuneus (Zhu et al., 2012).

In another study of the same sample by Zhu et al., 11 pre-defined ROIs were used in order to assess in more detail the connectivity within the DMN and revealed increased FC within the DMN (Zhu et al., 2017). Finally, in a very large study (Yan et al., 2019), DMN connectivity in cohort of 318 first-episode drug-naïve patients no significant changes were found in DMN connectivity when that group was compared to HCs (n = 266). Interestingly, when the treatment-naïve patients were compared to medicated first-episode MDD patients, decreased DMN connectivity was found in the treatment group. A summary of those studies based on the connectivity methodology that they have used is also available in Table 1.

### Acute effects of ketamine administration on brain connectivity – healthy volunteers

#### Subcortical ROIs

The acute effects of ketamine on fronto-striato-thalamic connectivity with the rest of the brain, revealed that ketamine, compared to placebo, increased the connectivity (n = 21) between the dorsal caudate and the thalamus bilaterally, as well as the ventral striatum and the superior and inferior ventromedial prefrontal cortex and the frontopolar cortex (Dandash et al., 2015). Ketamine-induced increases in the connectivity correlated with the changes in positive psychosis symptoms as well as the dissociative effects that accompany acute ketamine administration.

#### Cortical ROIs

Four studies have used cortical seeds to investigate the acute effects of ketamine’s administration in brain connectivity. Specifically, when the connectivity between the dorsal lateral PFC and the hippocampus was examined, ketamine increased the connectivity between the dorsal lateral PFC (left and right) and the left hippocampus (Grimm et al., 2015) in a sample of 24 healthy participants. When cortico-limbic connectivity was examined (n = 23) between the subgenual Anterior Cingulate Cortex (sgACC) and the hippocampus and the amygdala, no significant changes were identified between the ketamine and placebo sessions (Scheidegger et al., 2016). Moreover, in a study by Anticevic and colleagues (n = 19), Global Brain Connectivity (GBC) was used to study connectivity of the PFC and voxels within that region and increased connectivity was found between the superior frontal gyrus and the middle frontal gyrus (Anticevic et al., 2015) in ketamine compared to placebo.

The acute effects of ketamine’s administration on corticohippocampal connectivity were examined by Khalili-Mahani et al. (2015). In this study of 12 participants, the hippocampus was segmented into three anatomical regions: body, head and tail and the connectivity of those regions was correlated with the rest of the brain. Acute ketamine administration increased the connectivity between the hippocampal body (bilateral) and the superior part of the precuneus, the premotor cortex and the lateral visual cortices, compared to placebo (Khalili-Mahani et al., 2015). Hippocampal connectivity under ketamine was also examined (n = 19) focusing on the left hippocampus and decreased connectivity between that seed region and several brain areas including the ACC, the PCC and the insula (Kraguljac et al., 2017) was identified. Finally, Zacharias et al., have used the PCC/precuneus as a seed region to examine the connectivity of the DMN with the rest of the brain in a sample of 24 healthy volunteers. Ketamine compared to placebo increased the connectivity between the seed region and the medial Prefrontal Cortex (mPFC). Decrease connectivity was observed between the PCC/precuneus and the interparietal lobe, bilaterally (Zacharias et al., 2019).

#### Networks

The majority of studies – seven out of fifteen – that examined the effects of acute ketamine administration on connectivity focused on brain networks. Bonhomme and colleagues assessed FC changes (n = 14) induced by different doses of ketamine in brain networks related to consciousness including the DMN, the right and left executive control network, the salience network, the auditory network, the sensorimotor network and visual network. Ketamine, when administered in doses that were relevant for this review, reversed the significant anticorrelations that were identified between the DMN and three brain clusters (see Table 2). Moreover, within the DMN, ketamine produced a breakdown of connectivity and there was a significant correlation between the depth of sedation and decreased connectivity of the mPFC with the DMN (Bonhomme et al., 2016).

**Table 2.** Studies investigating changes in brain connectivity after acute ketamine administration in healthy volunteers are summarised in this table. The methodology, aims and hypotheses as well as a brief description of the main findings are included for each study.

Acute effects of ketamine’s administration on resting-state fMRI in healthy volunteers					
GBC (Global Brain Connectivity)					
Study	Subjects	Methodology			Summary of results
		Infusion protocol	Connectivity analysis	Aims/hypotheses	
Anticevic et al. (2015)	Pharmacological imaging HC, n = 19 males. Early Course and Chronic Schizophrenia, High Risk and Healthy Control Comparison EC-SCZ, n = 28. C-SCZ, n = 20. HR, n = 21. HC, n = 96	Intravenous administration of racemic ketamine via bolus (0.23 mg/kg over 1 min) followed by continuous infusion (0.58 mg/kg over 1 h).	GBC focusing only on the PFC and voxels within that region.	Acute ketamine administration would be associated with PFC functional hyperconnectivity. Explore the differences in PFC connectivity across schizophrenia stages. Ketamine’s effects would resemble early course but not chronic schizophrenia.	Pharmacological imaging Ketamine > Placebo L/R Superior Frontal Gyrus and R Middle Frontal Gyrus. No regions showed connectivity decrease following ketamine administration. Comparison of early course (EC-SCZ) and chronic schizophrenia (C-SHZ), high risk (HR) and healthy control (HC). Right Superior Lateral Prefrontal Cortex HC > C-SCZ and EC-SCZ > C-SCZ Superior Medial Prefrontal Cortex EC-SCZ > HC, HR > HC, EC-SCZ > C-SCZ Ketamine’s effects on PFC connectivity appear to be more relevant to earlier than later stages of schizophrenia.
Driesen et al. (2013)	HC, n = 22 (14 males).	Intravenous administration of racemic ketamine via bolus (0.23 mg/kg over 1 min) followed by continuous infusion (0.58 mg/kg over 1 h).	GBC analysis of the whole brain – correlation with positive, negative and cognitive symptoms as captured by the PANSS.	Acute ketamine administration would alter cortical functional connectivity during rest and that would relate to psychosis symptoms.	GBC analysis Ketamine > Placebo Increase in connectivity occurred across all voxels in the brain and no discrete clusters of increased GBC were identified within this pattern. Increased GBC under ketamine in the: L/R Paracentral Lobule, L/R Posterior Areas, L Middle Occipital Gyrus, L Parietal Operculum, L Insula, L Precentral Gyrus, L Medial Frontal Gyrus, R Middle Frontal Gyrus predicts positive symptoms. Increased GBC under ketamine in the: Dorsal Anterior Striatum, Medial Anterior Striatum and Thalamus predicts negative symptoms. No correlations were found between changes in GBC and cognitive symptoms.
ROI-to-ROI analysis					
Study	Subjects	Methodology			Summary of results
		Infusion protocol	Connectivity analysis	Aims/hypotheses	
Grimm et al. (2015)	HC, n = 24 (12 males). Rats, n = 9.	Humans Steady-State intravenous ketamine infusion of 0.5 mg/kg. Resting-State scanning 20-min post-infusion. Animals Subcutaneous injection of S-ketamine.	Humans ROI-to-ROI connectivity analysis between the dorsal lateral PFC and the hippocampus bilaterally. Animals ROI-to-ROI connectivity analysis between the left and right prelimbic cortex and the hippocampus bilaterally.	Acute ketamine administration would increase the PFC-HC connectivity in the human and rat brain.	Humans Ketamine > Placebo Right dorsal lateral PFC and left Hippocampus and Left dorsal lateral PFC and left Hippocampus. Animals Ketamine > Placebo Left Prelimbic Cortex and left Hippocampus, Left Prelimbic Cortex and right Hippocampus and Right Prelimbic Cortex and left Hippocampus.
Scheidegger et al. (2016)	HC, n = 23 (12 males).	Intravenous administration of S-ketamine via bolus (0.12 mg/kg) followed by continuous infusion (0.25 mg/kg/h).	ROI-to-ROI cortico-limbic connectivity Seed regions include the bilateral pregenual ACC (Anterior Cingulate Cortex), the bilateral hippocampus and the bilateral amygdala. Percentage changes in the BOLD signal during an emotional faces task were correlated with cortico-limbic connectivity changes.	Ketamine infusion would decrease reactivity in the amygdalo-hippocampal complex, during processing of negative stimuli. This reduction would be reflected in changes in functional connectivity to the pregenual ACC.	Cortico-limbic Connectivity analysis No significant changes in cortico-limbic functional connectivity between ketamine and placebo. BOLD change signal correlations During Ketamine administration: % BOLD signal changes to negative pictures positively correlated with functional connectivity to the pregenual ACC and bilateral Amygdala. No significant correlation between % BOLD change for positive or neutral stimuli and connectivity changes.
ROI to whole brain					
Study	Subjects	Methodology			Summary of results
		Infusion protocol	Connectivity analysis	Aims/hypotheses	
Zacharias et al. (2019)	HC, n = 24 males.	Intravenous administration of S-ketamine (0.1 mg/kg) for 5 min. Infusion stopped for 1 min and continued at 0.015625 mg/kg/min for a maximum of 1 h with a 10% reduction in the dose every 10 min.	Functional connectivity of the Default Mode network with the rest of the brain was assessed using the PCC/precuneus as a core region.	Frontal decrease of DMN functional connectivity to parietal brain regions.	Default Mode Network Connectivity Ketamine < Placebo PCC/precuneus – Medial Prefrontal Cortex. Ketamine > Placebo PCC/precuneus – Left and Right Interparietal Lobe.
Bonhomme et al. (2016)	HC, n = 14 (9 males). Six excluded from the analysis.	Intravenous administration of racemic ketamine following the Domino protocol. Ketamine target concentrations progressively increased by steps of 0.5 μγ/mL until deep sedation was achieved.	Functional Connectivity was assessed in specific networks which included: Default Mode Network (DMN). Right and Left Executive Control Network. Salience Network. Auditory Network. Sensorimotor Network. Visual Network. Networks were identified using specific ROIs and the activation in these regions was correlated to the rest of the brain. Resting-State data were acquired in the absence of ketamine, during light ketamine sedation and ketamine-induced unresponsiveness.	To explore the effects of ketamine at different anaesthetic doses on brain connectivity.	Default Mode Network Connectivity Ketamine > Placebo Cluster 1: Right Supramarginal gyrus, Bilateral Somatosensory cortex and Insula Cortex. Cluster 2: Bilateral Premotor Cortes, Ventral ACC and Dorsal ACC. Cluster 3: Left Supramarginal Gyrus, Somatosensory Association Cortex, Insular Cortex, Primary Auditory Cortex and Subcentral Area.
Dandash et al. (2015)	HC, n = 21 (10 males). Two excluded from the analysis.	Intravenous ketamine administration using a three-compartment pharmacokinetic model to achieve a standard plasma concentration of 100 ng/mL using a computerised pump.	Corticostriatal functional connectivity was characterised in relation to the following bilateral seed regions: Dorsal Caudate. Ventral Striatum/Nucleus Accumbens. Dorsal-Caudal Putamen. Ventral-Rostral Putamen. Changes in connectivity were also correlated with in positive psychotic symptoms (RSPS) and the brief rating scale (BPRS) as well as dissociative symptoms (CADSS)	Ketamine would reduce the functional connectivity in the dorsal fronto-striato-thalaminc circuit.	Corticostriatal Functional Connectivity Ketamine > Placebo Dorsal Caudate – L/R Thalamus and Ventral Striatum – L Superior Ventromedial Prefrontal Cortex, Ventral Striatum – L Frontopolar Cortex, Ventral Striatum – L Inferior Ventromedial Prefrontal Cortex. Correlation with psychosis symptom scales Higher ketamine-induced functional connectivity between: the L Ventral Striatum – L Superior Ventromedial Prefrontal Cortex and R Dorsal Caudate – R Midbrain. Correlated with higher ΔBPRS scores Higher ketamine-induced functional connectivity between: the L Ventral Striatum – L Inferior Ventromedial Prefrontal Cortex and L Dorsal Caudate – L Ventromedial Thalamus and Subthalamic Nuclei. Correlated with higher ΔCADSS scores Higher ketamine-induced functional connectivity between: the L Dorsal Caudate – L Ventrolateral Prefrontal Cortex and R Dorsal Caudate – R Midbrain.
Höflich et al. (2015)	HC, n = 35 (18 males). Five excluded from analysis.	Intravenous administration of S-ketamine (5 mg/mL) using a 1-min bolus of 11 mg/kg followed by a maintenance infusion of 0.12 mg/kg for 19 min.	Analysis 1 Seed-based analysis of the thalamus hub network including: L/R Thalamus. Cingulate Cortex. Lingual Gyrus. The time course of these seed regions was correlated with the entire brain. Analysis 2 Seed-based analysis of the cortico-thalamic network including: Motor cortex/Supplementary Motor Area. Somatosensory cortex. Temporal lobe. Posterior Parietal Cortex. Occipital Lobule.	To explore the involvement of specific functional connections of the thalamus in the schizophrenia-like state.	Analysis 1 Ketamine > Placebo Increased connectivity between the thalamus hub network and a bilateral cluster extending from the superior parietal lobule towards the temporal cortex, including post and precentral gyri. These changes start at 2.5-min post-infusion and remain for 17.5 min after the end of the infusion. Analysis 2 Ketamine > Placebo Increased connectivity of the post-central gyrus with the ventromedial region of the thalamus as well as the temporal seed region with the medial dorsal nucleus.
Khalili-Mahani et al. (2015)	HC, n = 12 male.	Intravenous administration of S-ketamine, 20 mg/70 kg/h for the first 60 min followed by 40 mg/70 kg/h for another 60 min.	The hippocampus was segmented into three anatomical regions: body, head, tail and the time series of these regions were correlated with the rest of the brain.	To investigate ketamine’s effects on the biomarkers of stress, including corticohippocampal connectivity.	Emergence of connectivity between the hippocampal head and the insula, medial visual and posterior parietal cortices Ketamine > Placebo L/R hippocampal body – superior part of the precuneus, L/R hippocampal body – premotor cortex and L/R hippocampal body – lateral visual cortices.
Niesters et al. (2012)	HC, n = 12 male.	Intravenous administration of S-ketamine, 20 mg/70 kg/h for the first 60 min followed by 40 mg/70 kg/h for another 60 min. Acquisition of data occurred during the last 10 min of the low-dose administration and the last 10 min of the high-dose administration.	Networks of Interest Medial Visual Network (NOI1). Lateral Visual Network (NOI2). Auditory-Somatosensory Network (NOI3). Sensorimotor Network (NOI4). Default Mode Network (NOI5). Executive Saline Network (NOI6). Visual-Spatial Network (NOI7). Working Memory Network (NOI8).	Resting-state fMRI would be able to detect ketamine-induced alterations in large-scale network patterns that would involve brain areas associated with analgesia, ketamine’s side effects and pain processing.	Medial Visual Network Connectivity Ketamine > Placebo NOI1: R Frontal Lobe, L Thalamus, R Primary Somatosensory cortex, L Secondary Somatosensory cortex, L Occipital cortex, L Optic radiation, R Supramarginal Gyrus, R Cerebellum. Auditory and Somatosensory Network Connectivity Ketamine > Placebo NOI3: R Hippocampus, L Precuneus, R Primary Visual Cortex, L Orbitofrontal Cortex, L Premotor Cortex, R Middle Temporal Gyrus, R Thalamus, L Primary Auditory Cortex, L/R Caudate Nucleus, L Anterior/Posterior Cingulate Cortex, L Lateral Occipital Cortex, L Amygdala, L Superior Longitudinal Fasciculus, R Insula, R Occipital Cortex and L Cerebellum.
Kraguljac et al. (2017)	HC, n = 19 male.	Intravenous bolus (0.27 mg/kg over 10 min) infusion, followed by a continuous infusion (0.25 mg/kg/h flow rate of 0.02 mL/s). Resting-state scans started 45 min after the start of the challenge and lasted for 7.5 min.	The left hippocampus was used as seed and time series from that regions were correlated with the rest of the brain.	Ketamine’s administration would result in fronto-temporal and temporo-parietal functional dysconnectivity.	Left hippocampal connectivity Ketamine < Placebo Left Hippocampus: Anterior Cingulate Cortex, Medial Prefrontal Cortex, Middle Cingulate, Bilateral Hippocampus, Right Insula, Posterior Cingulate Cortex, Lingual Gyrus and Calcarine Sulcus.
Mueller et al. (2018)	HC, n = 17.	Intravenous bolus infusion (0.1 mg/kg) of S-ketamine, followed by a continuous infusion of 0.015625 mg/kg/min for maximum 1 h with a 10% dosage reduction every 10 min.	Seeds were selected to represent five brain networks and their mean BOLD time series was correlated with the rest of the brain Seeds include: Posterior Cingulate Cortex for the Default Mode Network. Bilateral Intraparietal sulcus for the Dorsal Attention Network. Bilateral DLPFC for Executive Control Network. Bilateral fronto-insular cortex for the Salience Network. The PANSS and 5D-ASC were administered before and after ketamine and the delta score was correlated with changes in ketamine-induced changes in connectivity.	To examine ketamine’s effects on the Default Mode Network, the Dorsal Attention Network, the Executive Control Network and the Salience Network.	Executive Control Network Connectivity Analysis Ketamine < Placebo DLPFC and Bilateral Calcarine Fissure Ketamine > Placebo DLPFC and Left Anterior Cingulum and Left Superior Frontal Gyrus. Salience Network Connectivity Analysis Ketamine < Placebo Insular Cortex and Right Calcarine Fissure. Correlation with clinical symptoms Connectivity between the fronto-insular cortex (Salience Network) and the right calcarine fissure correlates with negative symptoms as captured by the PANSS.
Within network connectivity					
Study	Subjects	Methodology			Summary of results
		Infusion protocol	Connectivity analysis	Aims/hypothesis	
Spies et al. (2019)	HC, n = 35 (18 males). Five excluded from analysis.	Intravenous administration of S-ketamine (5 mg/mL) using a 1-min bolus of 11 mg/kg followed by a maintenance infusion of 0.12 mg/kg for 19 min.	Functional connectivity of within and between all main brain networks was assessed using different methodological approaches to evaluate the effect of esketamine on rsFC.	To assess the influence of ketamine on functional connectivity using multiple FC estimation methods.	Converging results between the different approaches show that Esketamine < Placebo Within the left Visual Network and between the left Visual Network and the right Visual Network.
Kleinloog et al. (2015)	HC, n = 5.	Intravenous administration of S-ketamine, 20 mg/70 kg/h for the first 60 min followed by 40 mg/70 kg/h for another 60 min. Acquisition of data occurred during the last 10 min of the low-dose administration and the last 10 min of the high-dose administration.	Within connectivity was examined in those networks: Medial Visual Network. Occipital Visual Network. Lateral Visual Network. Default Mode Network. Cerebellum Network. Sensorimotor Network. Auditory Network. Executive Control Network. Right Frontoparietal Network. Left Frontoparietal Network. Changes in connectivity were also correlated with subjective effects.	The connectivity within the DMN and specifically that of the posterior cingulate cortex would be associated with the psychotomimetic effects.	Within Network Connectivity Analysis Connectivity between the sensory motor network and a cluster containing the PCC and the Precentral Gyrus significantly correlated with subjective effects of perception under ketamine.
Pattern recognition connectivity networks					
Joules et al. (2015)	HC, n = 18.	Intravenous administration of racemic ketamine, 1 min bolus infusion of 0.12 mg/kg/h followed by a steady state 0.31 mg/kg/h.	Node connectivity and pattern recognition techniques	To identify spatial patterns of whole-brain connectivity underlying the effects of ketamine.	Node Connectivity Analysis Ketamine > Placebo Basal Ganglia and Cerebellum Ketamine < Placebo Occipital Cortex, Temporal Cortex, Medial Temporal Cortex and Frontal Cortex
Delayed effects of ketamine administration on resting-state fMRI in healthy volunteers					
ROI-to-ROI					
Study	Subjects	Methodology			Summary of results
		Infusion protocol	Connectivity analysis	Aims/hypothesis	
Lehmann et al. (2016)	HC, n = 17.	Steady-state infusion of 0.25 mg/kg of S-ketamine.	Connectivity between the posterior ACC (pACC) and dorsal PCC (dPCC) was assessed. The activation of these regions was also correlated with psychotomimetic effects as captured by the ‘5D-ASC’ scale. Participants were scanned 24 h after the ketamine/placebo administration.	The psychotomimetic experiences of ketamine might be related to changes in functional connectivity 24 h after its administration.	Ketamine < Placebo Reduced connectivity between the pACC and dPCC. Correlation with psychotomimetic changes The stronger the psychotomimetic effects, the more reduced the resting-state connectivity between the pACC and dPCC.
ROI to whole brain					
Scheidegger et al. (2012)	HC, n = 17.	Steady-state infusion of 0.25 mg/kg of S-ketamine.	ROI to whole-brain connectivity analysis was conducted using the following key network regions as seeds: Bilateral DLPFC – Cognitive Control Network. Bilateral PCC – Default Mode Network. sgACC – Affective Network.Connectivity between the dorsal nexus* and the whole brain was assessed.Participants were scanned 24 h after the ketamine/placebo administration.	To investigate pharmacological changes in functional connectivity in the healthy brain as a model for ketamine’s antidepressant actions.	Seed-Based Analysis Ketamine < Placebo Default Mode Network Connectivity of the Bilateral PCC with the Bilateral DMPFC, posterior ACC and mPFC. Affective Network Connectivity of the sgACC with the right DMPFC. Connectivity of the Dorsal Nexus with the PCC
Li et al. (2020)	HC, n = 61.	Steady-state infusion of 0.5 mg/kg of S-ketamine or saline.	Seed-based FC at the dPCC. fALFF to assess whole-brain activity changes from baseline to 1 and 24 h. Participants underwent two MRI sessions: Day 1: baseline scan (20 min prior to infusion) and 1 h after infusion. Day 2: 24 h post-infusion	A data-driven investigation of ketamine – induced effects at 1 and 24 h.	FC Results 1 h post-ketamine compared to placebo – no significant changes. 24 h post-ketamine compared to placebo Decreased FC between the dPCC and the DMPFC (strongest finding), the inferior frontal gyrus and the vMPFC and pgACC and increased FC between the dPCC and the precuneus. fALFF Results 1 h post-ketamine compared to placebo Increased fALFF in the ventral PCC and decreased fALFF in the bilateral inferior occipital gyri. 24 h post-ketamine compared to placebo – no significant changes. Post-hoc seed based analysis in the vPCC 1 h post-ketamine compared to placebo Decreased FC between the vPCC and the midcingulate cortex. 24 h post-ketamine compared to placebo – no significant changes.

* The dorsal nexus seed was created by overlapping voxels that showed significant changes in both the posterior and subgenual cingulate cortices.

fMRI: functional magnetic resonance imaging; PFC: prefrontal cortex; BOLD: blood oxygen level–dependent; PCC: posterior cingulate cortex; DMN: default mode network; fALFF: Fractional amplitude of low-frequency fluctuation.

When the connectivity of the thalamus hub network (n = 35) and the cortico-thalamic network were examined, significant increases were identified when acute ketamine was compared to placebo. Specifically, ketamine increased connectivity between the thalamus hub network and a cluster extending from the superior parietal lobule towards the temporal cortex. In the cortico-thalamic network, ketamine increased the connectivity of the post-central gyrus with the ventromedial region of the thalamus as well as the temporal lobe with medial dorsal nucleus (Höflich et al., 2015).

Niesters et al. (2012) showed that acute ketamine administration (n = 12) increased the connectivity between the medial visual network and the thalamus, the occipital cortex, the primary and secondary somatosensory cortex. In the same study, increase connectivity was also identified between the auditory and somatosensory network and brain regions including the hippocampus, the precuneus, the thalamus, the caudate nucleus, the anterior and PCC as well as the insula and cerebellum (for details, see Table 2) (Niesters et al., 2012). In a study by Spies et al., various methodological approaches were used to examine the acute effects of ketamine on brain connectivity (n = 35) compared to placebo. Decreased connectivity within the left visual network and between the left and right visual network was the common finding between the different methodologies (Spies et al., 2019).

Increases as well as decreases following acute ketamine administration (n = 17) were also identified when the FC between the executive control network and the salience network (SN) with the rest of the brain was examined and compared to placebo. The DLPFC was used as a seed region to examine connectivity between the executive control network and the rest of the brain, and it was shown that ketamine decreased the connectivity between the DLPFC and the bilateral calcarine fissure. Connectivity was increased, following ketamine administration, between the DLPFC and the left anterior cingulum and the left superior frontal gyrus. Decreased connectivity was identified between the insular cortex – a seed region for the SN – and the right calcarine fissure. This decrease in connectivity correlated with negative symptoms as captured by the PANSS (Mueller et al., 2018). A positive correlation between the sensory motor network and a cluster containing the PCC and subjective effects of perception under ketamine was also identified in another study examining the psychotomimetic effects of several pharmacological compounds including ketamine, this study however, had a very small sample size (n = 5) (Kleinloog et al., 2015).

#### Global connectivity and pattern recognition techniques

When GBC was assessed at a whole-brain level, ketamine (n = 22) produced a significant increase across all voxels in the brain (Driesen et al., 2013), compared to placebo. Moreover, increases in GBC in several cortical and subcortical brain areas predicted the presence of positive symptoms or the absence of negative symptoms as measured by the PANSS. Pattern recognition techniques were also used to compare ketamine and placebo (n = 18) and it was shown that ketamine produced a pattern of brain activations that could be discriminated from placebo with high accuracy (Joules et al., 2015). The nodes that appeared to have a strong presence in the pattern were subcortical nodes including the caudate, thalamus and cerebellum. These showed increased connectivity on ketamine, whereas the cortical nodes on average showed decreased connectivity on ketamine.

### Delayed effects of ketamine administration on brain connectivity – healthy volunteers

Two studies have investigated the delayed (24-h post-administration) effects of ketamine in healthy volunteers (n = 24) using resting-state fMRI. The first study examined the connectivity between the pACC and dPCC, and it was shown that 24-h post-ketamine connectivity reduced between those two brain areas. Moreover, the reduction in connectivity between these two areas correlated with increased acute psychotomimetic symptoms (Lehmann et al., 2016). The second study (n = 17) used as seeds key brain areas of the cognitive control network (bilateral DLPFC), the DMN (bilateral PCC) and the Affective Network (sgACC). The connectivity of the dorsal nexus with the rest of the brain was also examined. Ketamine decreased connectivity between the bilateral PCC (DMN seed) and the bilateral DMPFC, posterior ACC and mPFC, compared to placebo. Decreased connectivity was also identified between the sgACC (affective network seed) and the right DMPFC whereas the dorsal nexus presented with decreased connectivity with the PCC (Scheidegger et al., 2012). A summary of those studies can be found in Table 2.

### Antidepressant effects of ketamine administration on brain connectivity – MDD patients

Most studies investigating the effects of ketamine in MDD patients focus on 24 h past the drug administration, when ketamine’s antidepressant effects peak. Abdallah and colleagues found that before the ketamine administration, patients with MDD present with decreased GBC in the PFC compared to HCs. When connectivity was assessed 24-h post-ketamine administration, MDD patients presented with increased connectivity in the lateral PFC and reduced GBC in the left cerebellum. Moreover, when the MDD patients were classified as ketamine responders and non-responders, it was shown that responders had increased GBC in the bilateral caudate, the right lateral PFC and the left middle temporal gyrus (Abdallah et al., 2017).

Another study looked at the delayed effects of ketamine, compared to placebo, 2 days and 10 days after the ketamine administration on MDD patients and HCs. An ROI approach was used in order to assess connectivity changes in the SN, the central executive control network and the DMN. At baseline, MDD patients exhibited decreased connectivity between the DMN and the right dorsolateral PFC (BA6 and BA9) and the left postcentral gyrus (insula to BA43). When the effects of ketamine were compared to placebo, 48 h (day 2) after the drug administration, between MDD and HCs, smaller differences in the connectivity between the DMN and the insula were identified for the two groups which normalised at day 10. In contrast, the ACC showed increased connectivity in the HCs compared to MDDs at day 2 that was still apparent at day 10 and was not present at baseline scans or the placebo session (Evans et al., 2018).

Using the same data set, Kraus and colleagues used GBC in order to assess connectivity changes in MDD patients compared to HCs and examine the effects of ketamine. At baseline, MDD patients had decreased GBC at the middle Cingulate Cortex (MCC) and the ACC. When the GBC was examined specifically at the PFC, it was shown that at baseline MDD patients had decreased connectivity in the right superior frontal cortex and the right middle frontal cortex. However, no significant changes were observed between the ketamine and placebo sessions in MDD patients (Kraus et al., 2020). The same data set was also used to assess the effects of ketamine on striatal connectivity. It was shown that 2 days after the ketamine administration, MDD patients presented with increased connectivity between the different striatal seeds and areas of the prefrontal cortex, ACC and OFC. Decreased connectivity was identified, after ketamine in healthy volunteers. Increased connectivity between the dorsal caudate and the ventromedial prefrontal cortex, 2 days and 10 days after ketamine, also correlated with a reduction in anhedonia in the MDD group (Mkrtchian et al., 2020).

Finally, in a study by Vasavada et al., the effects of four ketamine infusions on the connectivity of the amygdala and the hippocampus with the Central Executive Network (CEN), the DMN and the SN were examined and compared to placebo. Decreased connectivity between the amygdala and the left CEN as well as increased negative connectivity between the right hippocampus and the left CEN was identified when connectivity was assessed 24 h after the first infusion and was compared to baseline. This increase in hippocampal connectivity predicted decreases in anhedonia. When the effects of ketamine on connectivity were assessed 24–72 h after the fourth infusion and were compared to baseline, ketamine increased the connectivity between the amygdala and the right CEN as well as the negative connectivity between the right hippocampus and the left CEN and decreased the connectivity between the left amygdala and the SN and this change predicted improvements in anxiety (Vasavada et al., 2021). A summary of those studies can be found in Table 3.

**Table 3.** Acute and delayed (2 h to 2 weeks post-drug administration) changes in brain connectivity after ketamine administration in MDD patients are summarised in this table. The studies are classified based on their connectivity methodology and a brief description of the aims, hypotheses and main findings are included for each study.

Delayed effects of ketamine’s administration on resting-state fMRI in depressed volunteers					
Single dose					
GBC (Global Brain Connectivity)					
Study	Subjects	Methodology			Summary of results
		Infusion protocol	Connectivity analysis	Aims/hypothesis	
Kraus et al. (2020)	MDD, n = 33. HCs, n = 22. Patients were treatment-resistant and in a current depressive episode at the time of the scan. All patients were medication free for 2 weeks before randomisation and for the duration of the study.	Intravenous administration of racemic ketamine (0.5 mg/kg over 40 min) via a steady-state, continuous infusion.	GBC analysis of the whole brain as well as intra-PFC GBC. Participants were scanned at baseline and on day 2 and day 10 after the ketamine and placebo infusion.	To independently replicate the finding of disrupted GBCr in individuals with MDD. to examine the specific effects of pre-processing strategies on GBCr.	Whole-brain GBC at baseline MDD < HC Bilateral MCC and ACC spreading to the superior medial frontal cortices. Intra-PFC GBC at baseline MDD < HC Right superior frontal cortex and Right middle frontal cortex. No effects of ketamine vs placebo were observed in GBCr, 24-h post-ketamine administration.
Abdallah et al. (2017)	MDD, n = 18. HC, n = 25. Patients had a chronic and treatment-refractory illness. Following ketamine, 56% of MDD patients achieved response.	Intravenous administration of racemic ketamine (0.5 mg/kg over 40 min) via a steady-state, continuous infusion.	GBC analysis of the whole brain. Participants received a baseline rs-fMRI scan. MDD patients underwent a repeated rs-fMRI scan 24-h post-ketamine infusion.	Patients with MDD in a current depressive episode will show reduced PFC GBCr. Mood normalisation, following ketamine treatment, would parallel a normalisation in the functional connectivity.	MDD group-pre ketamine Widespread dysconnectivity in MDD compared to HC in the PFC. MDD group post-ketamine Ketamine increased GBCr in the lateral PFC and reduced GBC in the left cerebellum. MDD group post-ketamine Responders > non-responders Bilateral Caudate, Right lateral PFC and Left middle Temporal Gyrus.
ROI to whole brain					
Study	Subjects	Methodology			Summary of results
		Infusion protocol	Connectivity analysis	Aims/hypothesis	
Mkrtchian et al. (2020)	See Evans et al., 2018.	See Evans et al., 2018.	ROI analysis of bilateral striatal subregions including: Ventral Striatum (VS). Dorsal Caudate (DC). Dorsal Caudate Putamen (DCP). Ventral-Rostral Putamen (VRP). Inflammatory biomarkers (CRP), anhedonia (SHAPS) and depression scores (MADRS) were examined in relation to connectivity changes.	Ketamine would increase functional connectivity within the fronto-striatal circuitry of Treatment Resistant Depressed (TRD) participants but decrease in HVs. These effects would be associated with ketamine-induced changes in inflammatory response.	HCs vs MDD Baseline compared to Day 2, increased connectivity after ketamine VS – left dorsolateral Prefrontal Cortex, DC – right ventrolateral Prefrontal Cortex, DCP – pregenual Anterior Cingulate Cortex and VRP – Orbital Frontal Cortex. Baseline compared to Day 2, ketamine decreased connectivity in the same brain areas as in the MDD group, described above. Relationship between connectivity changes and CRP: Increased CRP levels correlated with decreased connectivity between the VRP–Orbital Frontal Cortex, in HCs. Relationship between connectivity changes and SHAPS scores on Day 2: Reduction in SHAPS scores correlated with increased connectivity between the DC – right ventrolateral Prefrontal Cortex, post-ketamine in the MDD group. Relationship between connectivity changes and SHAPS scores on Day 10: Reduction in SHAPS scores correlated with post-ketamine increases in the connectivity between the DC – right ventrolateral Prefrontal Cortex in the MDD group.
Evans et al. (2018)	MDD, n = 33.HC, n = 25.Patients were treatment-resistant and experiencing a depressive episode at the time of the scan.All patients were medication free for 2 weeks before randomisation and for the duration of the study.Following ketamine administration depression scores were reduced for MDD patients and remained significantly improved for 2 days post-infusion.	Intravenous administration of racemic ketamine (0.5 mg/kg over 40 min) via a steady-state, continuous infusion.The study was a placebo-controlled, cross over design.	ROI analysis of the Salience Network (SAL). Central Executive Network (CEN). Default Mode Network (DMN).Participants were scanned at baseline and on Day 2 and Day 10 after the ketamine and placebo infusion.	DMN differences between the MDD and HC subjects would be reduced after ketamine administration, particularly in regions associated with SAL and CEN.	HCs vs MDD Baseline Right dorsolateral PFC (BA6 and BA9) and Left postcentral gyrus (insula to BA43).Baseline, Day 2 and Day 10, both for increased DMN connectivity with: Right precentral gyrus and Bilateral post-central gyrus for both the ketamine and placebo sessions. Day 2 of the ketamine session Smaller difference between the HCs and MDD in connectivity between the DMN and the insula which normalised at Day 10. The ACC showed increased connectivity in the HCs compared to MDDs that was still apparent at Day 10 but not present during the Baseline scan or the placebo Day 2 scan. Day 10 of the ketamine session Increased connectivity of the right supramarginal gyrus (BAs 22 and 39) in subjects with MDD compared to HCs.Ketamine Day 2 > Placebo Day 2 MDD group Right and left insula, the Middle frontal gyrus (BA31), Post-central gyrus (BA5) and the Occipital gyrus (BAs 18 and19).HC group Left thalamus, the Cingulate cortex (BA24), the Cuneus (BA18) and the Right middle frontal gyrus (BAs 6, 8 and 9).Ketamine day 10 < Placebo Day 10 MDD group Occipital gyrus and Left dorsolateral prefrontal cortex (BA 9).Ketamine Day 10 < Placebo Day 10 MDD group Right post-central gyrus (BA 40).
Repeat dose					
ROI to network					
Study	Subjects	Methodology			Summary of results
		Infusion protocol	Connectivity analysis	Aims/hypothesis	
Vasavada et al. (2021)	MDD, n = 44. HC, n = 50. Patients were treatment-resistant and experiencing a depressive episode at the time of the scan. All patients were allowed to remain on stable antidepressant medication (unchanged for 6 weeks prior to scanning). Depressive scores significantly decreased after the four ketamine infusions.	Intravenous administration of racemic ketamine (0.5 mg/kg over 40 min) via a steady-state, continuous infusion. The study was a placebo-controlled, cross-over design. MDD patients received four ketamine infusion in 2–2.5 weeks. This study employed an open label experimental design.	Two bilateral ROIs were selected: Amygdala. Hippocampus. And the connectivity of the ROI with the: Default Mode Network. Central Executive Network. Salience Network was examined. MDD patients we scanned at: Baseline (T1-44 patients). 24 h post the first infusion (T2-43 patients) 24–72 h post the fourth infusion (T3-39 patients). HCs were scanned at: Baseline (T1-30 participants). 2 weeks after the first scan – 17 participants.	Functional connectivity between the amygdala and/or hippocampus and cortical RSNs would be deficient in depression and restored by ketamine in patients with TRD. Post-hoc analyses investigated cross-sectional differences between patients and control subjects and correlations with longitudinal change in FC in order to understand the antidepressant response under ketamine.	MDD vs HCS Baseline Increased connectivity between the right amygdala and right CEN in HCs compared to MDD patients. No changes in hippocampal connectivity between HCs and MDD patients. MDD group Ketamine T2 compared T1 Decreased connectivity of amygdala to left CEN. Increased negative connectivity of right hippocampus to left CEN. Ketamine T3 compared to T1 Increased connectivity of right amygdala to right CEN. Decreased connectivity of left amygdala with SN. Increased negative connectivity of right hippocampus to left CEN. Correlation of connectivity changes with symptoms’ improvement Acute change in functional connectivity between the left amygdala and the Salience Network after the first infusion correlated with post-treatment improvement in the BIS (Behavioural Inhibition System) scale. The same effect was observed at the end of the treatment. Acute change in functional connectivity between the hippocampus and the right Central Executive Network correlated with changes in the SHAPS (Snaith–Hamilton Pleasure Scale) scale after the first infusion.

## Discussion

### Drug-naïve MDD connectivity research

The studies that investigate connectivity changes in MDD drug-naïve patients (Table 1) can be divided into two groups. The first group includes studies that examine brain regions which are important for emotional regulation and cognition such as the hippocampus, fronto-limbic areas, the caudate and the NA (Cao et al., 2012; Gong et al., 2017; Yang and Wang, 2017). The second group comprises of only two studies that look at the DMN connectivity by examining this network as a whole (Zhu et al., 2012) and by selecting DMN seeds for connectivity analyses (Zhu et al., 2017).

In order to examine the brain regions that are important for emotional regulation, which appears to be problematic in depression and investigate how their connectivity might differ between patients and HCs, most studies have used seed-to-whole-brain connectivity approaches. The findings of these studies demonstrate that the ventral caudate and the superior temporal gyrus, which are often associated with emotional regulation and reward processing presented with increased connectivity with the occipital lobe and the precuneus (Yang and Wang, 2017) whereas the NA, also involved in reward processing, showed increased connectivity with several brain areas including the bilateral caudate, the medial OFC and the rostral ACC (Gong et al., 2017). The insula, however, a key region for interoceptive awareness and emotional regulation (Namkung et al., 2017), presented with decreased connectivity with brain regions that are part of the fronto-limbic circuitry (Guo et al., 2015). Some of these areas where connectivity changes have been identified such as striatal areas and the ACC present with lower glucose metabolism (Su et al., 2014; Wei et al., 2016) as measured by PET in patients with MDD indicating that these brain areas are promising targets for pharmacological agents such as ketamine that alter glutamatergic signalling.

When DMN connectivity was examine and compared between treatment-naïve individuals and HC, increases were identified between the different DMN subsystems as well as between the DMN and the rest of the brain (Zhu et al., 2017). Increased DMN activations have been linked to increased rumination, a very prominent characteristic of depression which persists in remission. Increased rumination is associated with overgeneral memory recall and a bias towards negative affect and is not currently targeted successfully by commonly prescribed antidepressants (Jones et al., 2008). It is striking, however, that only two of the thirteen studies identified in treatment-naïve depressed individuals have reported significant changes in DMN connectivity. In the study by Yan et al., when a very large sample of first-episode, treatment-naïve individuals were compared to HC, connectivity within the DMN did not differ between the two groups. Most interestingly, when DMN connectivity was compared between the first-episode, treatment-naïve patients and first-episode patients receiving treatment at the time of the scan, it was shown that the medicated patients exhibited significantly reduced connectivity. This result could indicate that some of the previous research findings about the DMN showing significantly altered connectivity between depressed patients and HCs could partly be attributed to antidepressant treatment (Yan et al., 2019). The long-term and short-term effects of antidepressant treatment on brain connectivity are not very well-understood and could, along with methodology inconsistencies, explain why in this review there in no great overlap between the brain areas that present with altered connectivity in treatment-naïve depressed individuals and the treatment-resistant depressed patients who are recruited in the ketamine studies when they are compared to control individuals. It is thus crucial for research studies to properly report the medication status of their participants.

### Ketamine’s effects in healthy volunteers

#### Acute ketamine effects in healthy volunteers

In general, acute ketamine administration in healthy volunteers caused an overall increase in brain connectivity (Anticevic et al., 2015; Driesen et al., 2013). This was a consistent finding despite the different methodologies used by different studies. An overall increase in brain connectivity during the ketamine infusion (acute ketamine administration) was associated the positive and negative symptoms psychosis-like symptoms produced by the drug. Changes in brain connectivity have been linked to changes in synaptic plasticity (Stampanoni Bassi et al., 2019) and might be necessary for the initiation of mechanisms – at the brain and neuronal level – that would mediate the antidepressant effects of the drug.

When brain networks were examined separately, acute ketamine administration produced changes in the connectivity of the DMN, the executive control network as well as networks that play an important role in the initiation of sedation and unconsciousness. The increased connectivity of these networks under acute ketamine administration could be associated with the anaesthetic effects of the drug seen at higher doses. However, several studies that specifically focused on connectivity changes within the DMN network, a network that is particular interest to depression, failed to report any significant differences after acute ketamine administration (Kleinloog et al., 2015; Mueller et al., 2018; Niesters et al., 2012).

Within the group of regions associated with emotional regulation and cognition and presented with increased connectivity under ketamine, the hippocampus has been the most examined. Research studies have identified increases in hippocampal connectivity with several brain regions including the insula, the precuneus, and the parietal cortex after ketamine administration compared to placebo (Grimm et al., 2015). This increase in hippocampal connectivity has been linked with ketamine’s effects on Long-Term Potentiation (Yan et al., 2019) and synaptic plasticity, a candidate mechanism for ketamine’s antidepressant action (Kavalali and Monteggia, 2020). Moreover, since the hippocampus mediates cognitive and spatial processing functions, this altered connectivity of the hippocampus could offer a potential explanation for the cognitive impairments that are observed during acute ketamine administration (Corlett et al., 2007).

#### The delayed and antidepressant effects of ketamine on brain connectivity

The antidepressant effects of ketamine are detectable 2 h after the administration of the drug, peak at 24-h post-infusion, and could last up to 1 week after a single ketamine infusion (REF). Ketamine’s antidepressant action, if mirrored in the brain as a normalisation of the changes in connectivity observed in depression, would be expected to reverse some of the changes in connectivity observed between MDD and HCs.

The studies that have looked at the antidepressant effects of ketamine on depressed individuals have shown that the decreased connectivity that was identified during the placebo session was reversed by ketamine. Specifically, GBC was found reduced in MDD compared to healthy participants that was increased 24-h post-ketamine administration (Abdallah et al., 2017); this finding, however, failed replication (Kraus et al., 2020). The drug effects on brain connectivity seem to persist even longer than 24 h since the decrease in the connectivity between the DMN and the insula that was identified for MDD patients compared to HCs was smaller 48 h after ketamine administration but returned to baseline 10 days past the ketamine infusion (Evans et al., 2018). Ketamine produced increases in the connectivity of striatal regions (Mkrtchian et al., 2020) with the dorsolateral and ventrolateral PFC, the pregenual ACC and the OFC in depressed individuals, while decreases were observed for the same set of brain regions in healthy volunteers. Moreover, the increases and decreases in the connectivity of the amygdala and the hippocampus also lasted for more than 24 h and were detectable after multiple ketamine infusion (Vasavada et al., 2021).

In healthy volunteers, however, our literature search revealed that 24-h post-ketamine administration, the connectivity of the DMN decreased compared to the placebo session (Scheidegger et al., 2016) and the connectivity between the pACC and dPCC also decreased 24-h post-ketamine (Lehmann et al., 2016). This potentially differential effect that the drug produces in depressed and healthy participants is rather interesting since it indicates that ketamine’s antidepressant effects could be specific to networks and deficits that are present in MDD and could be associated with neurotransmitter deficits that are observed in depression.

Several studies have looked at the delayed effects of ketamine, 2–24 h post-administration in depressed individuals and have used PET imaging to assess changes in glucose metabolism. Most of the findings of those studies show effects in limbic areas such as the amygdala (increased glucose metabolism post-ketamine) (Carlson et al., 2013) and the hippocampus (decreased glucose metabolism post-ketamine) (Nugent et al., 2014), brain areas that present with altered connectivity in depression and are thus potential targets for pharmacological modulation. Although only two studies so far have looked at the effects of ketamine on the striatum (Mkrtchian et al., 2020), the amygdala and the hippocampus (Vasavada et al., 2021), the fact that the drug produces detectable and long-lasting changes in the connectivity of these areas is rather promising, Some of these changes also correlate with improvements in anhedonia and anxiety and further underlie the importance of the glutamatergic modulation of these brain areas for the antidepressant effects of the drug.

## Limitations

Most of the studies that have looked at the acute effects of ketamine in healthy volunteers have investigated ketamine as a model of psychosis. In order to validate their hypotheses, these studies have selected to focus on brain regions that have been previously identified in research as important for psychosis. These regions, however, do not necessarily overlap with the brain areas that are key to the understanding of depression. Trying to reconcile the findings of the acute ketamine connectivity studies with those of depression was therefore challenging. In addition, these studies often use bolus-infusion techniques for ketamine administration, where the dose and duration of the infusion vary from study to study, whereas when used as an antidepressant, a slow infusion is given, usually over 40 min. The precise impact of these different infusion regimens has not been directly compared, but it seems the strength of the psychotomimetic effects may be reduced with the slower infusion (Ballard and Zarate, 2020).

Moreover, our stringent inclusion criteria aiming to bypass the confounding effects of antidepressant medication on brain structure (Dusi et al., 2015) greatly limited the number of studies included in our review and so we decided not to exclude any studies based on a sample size criterion. This mainly impacted the HC studies where there were a number of smaller studies (e.g. n = 12, 14, 17 and 18). Small sample sizes in neuroimaging and especially pharmacology studies that involve healthy volunteers have been a great concern in the field and could obscure true finding. In addition, different connectivity methodologies have been used to examine the differences in brain connectivity between healthy individuals and treatment-naïve depressed patients as well as the acute and delayed effects of ketamine and this would also be a confounding factor that could potentially explain inconsistencies in the results of these studies.

Most of the studies with drug-naïve depressed patients that have been included in our review recruited either first-episode depressed participants or patients with MDD without any history of treatment. The number of studies that failed to explicitly identify whether the patients received treatment or not was also striking. The majority of those studies were also conducted with an exclusively Chinese sample perhaps limiting the generalisation of these findings. Furthermore, no follow-up studies that we are aware of have looked at treatment response in those samples. We could thus assume that those studies consist of both treatment responders as well as treatment-resistant patients. The resting-state studies that have looked at ketamine as an antidepressant mainly focus on treatment-resistant patients since the clinical efficacy of ketamine has been most studies in these cases. The putative neuronal differences characterising these sub-groups are currently unknown. Whether ketamine would have a differential effect on responders and non-responders to conventional antidepressant treatment requires further investigation.

Finally, there is evidence that in pharmacological MRI and especially with NMDA receptor antagonists (acute ketamine administration), the tight relationship between the neuronal activity and regional blood flow is disrupted (Golanov and Reis, 1996; Långsjö et al., 2004). This makes the interpretation of the results of acute ketamine studies challenging since they could, at least partly, be attributed to the vascular effects of the drug (Iannetti and Wise, 2007). However, it has been shown that subanaesthetic doses of ketamine, as the ones used in the studies included in this review, do not produce a disturbed coupling between cerebral blood flow and metabolism (Långsjö et al., 2004, 2005). In addition, ketamine has been shown to induce primarily focal and task-dependent BOLD changes (Duncan et al., 1999; Littlewood et al., 2006) further supporting the idea that the connectivity changes observed under ketamine in our review are not the result of vascular changes but actual changes that the drug produces in neuronal activity.

## Conclusion

The aim of this review was to summarise the connectivity changes in treatment-naïve MDD patients and link these changes with the acute and delayed effects of ketamine in the brain connectivity of healthy and depressed individuals in order to understand how ketamine might exert its antidepressant actions. The very limited number of studies in treatment-naïve MDD patients along with the absence of any studies around the acute effects of ketamine in depressed patients and the only now emerging literature around the delayed effects of the drug make it difficult to draw robust conclusions about the mechanism of ketamine’s antidepressant actions and how this mechanism could relate to the changes in brain connectivity observed in depression in the absence of treatment. Some preliminary conclusions could be drawn through some of the consistent findings that have emerged. Treatment-naïve MDD patients exhibit an increased connectivity in reward and emotional processing areas including the striatum, the amygdala and the insula. Acute ketamine administration, in healthy volunteers, increases brain connectivity, at the network level as well as when single brain areas are examined, such as the hippocampus. These findings could be linked to increased synaptic plasticity that is produced by ketamine and is a candidate mechanism for the drug’s antidepressant actions. The delayed effects of ketamine’s administration (24 h to 10 days post-infusion) include increases in the connectivity of the striatum with frontal brain areas, leading to a normalisation of the connectivity differences between depressed individuals and HCs. The ketamine-induced increases in striatal connectivity could explain the anti-anhedonic effects of the drug, that might be mediated by changes in synaptic plasticity. These increases, however, are in contrast to the decreased striatal connectivity observed in treatment-naïve MDD individuals which might be linked to the reward-related deficits observed in these patients.
